# Autophagy drives osteogenic differentiation of human gingival mesenchymal stem cells

**DOI:** 10.1186/s12964-019-0414-7

**Published:** 2019-08-19

**Authors:** Chiara Vidoni, Alessandra Ferraresi, Eleonora Secomandi, Letizia Vallino, Chiara Gardin, Barbara Zavan, Carmen Mortellaro, Ciro Isidoro

**Affiliations:** 1 Laboratory of Molecular Pathology, Department of Health Sciences, Università del Piemonte Orientale “A. Avogadro”, Via P. Solaroli 17, 28100 Novara, Italy; 2 Maria Cecilia Hospital, GVM Care & Research, via Corriera 1, 48033, Cotignola, Ravenna, Italy; 3 Medical Sciences Department, University of Ferrara, Via Fossato di Mortara, 70 Ferrara, Italy; 4 Oral Surgery Unit, Department of Medical Science, Università del Piemonte Orientale “A. Avogadro”, Novara, Italy

**Keywords:** AMPK, BECLIN-1, Phytotherapy, Osteoblast, Resveratrol

## Abstract

**Background/aim:**

Autophagy is a macromolecular degradation process playing a pivotal role in the maintenance of stem-like features and in the morpho-functional remodeling of the tissues undergoing differentiation. In this work we investigated the involvement of autophagy in the osteogenic differentiation of mesenchymal stem cells originated from human gingiva (HGMSC). METHODS: To promote the osteogenic differentiation of HGMSCs we employed resveratrol, a nutraceutical known to modulate autophagy and cell differentiation, together with osteoblastic inductive factors. Osteoblastic differentiation and autophagy were monitored through western blotting and immunofluorescence staining of specific markers.

**Results:**

We show that HGMSCs can differentiate into osteoblasts when cultured in the presence of appropriate factors and that resveratrol accelerates this process by up-regulating autophagy. The prolonged incubation with dexamethasone, β-glycerophosphate and ascorbic acid induced the osteogenic differentiation of HGMSCc with increased expression of autophagy markers. Resveratrol (1 μM) alone elicited a less marked osteogenic differentiation yet it greatly induced autophagy and, when added to the osteogenic differentiation factors, it provoked a synergistic effect. Resveratrol and osteogenic inductive factors synergistically induced the AMPK-BECLIN-1 pro-autophagic pathway in differentiating HGMSCs, that was thereafter downregulated in osteoblastic differentiated cells. Pharmacologic inhibition of BECLIN-1-dependent autophagy precluded the osteogenic differentiation of HGMSCs.

**Conclusions:**

Autophagy modulation is instrumental for osteoblastic differentiation of HGMSCs. The present findings can be translated into the regenerative cell therapy of maxillary / mandibular bone defects.

**Graphical abstract:**

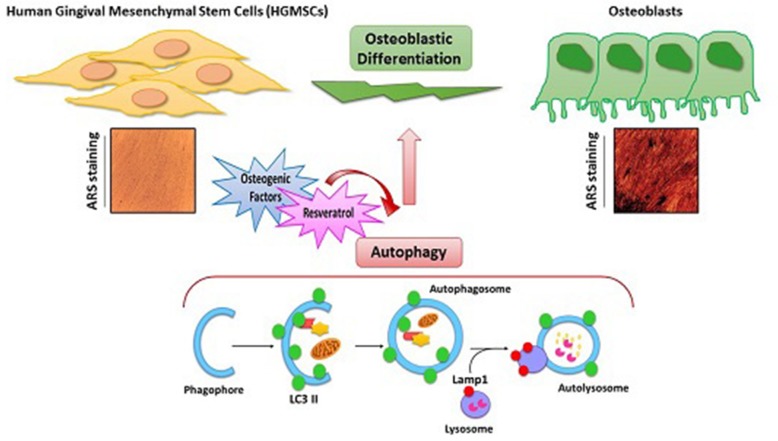

## Background

Bone resorption, bone wound healing and osteo-integration of implants remain major clinical challenges in orthopedics and dentistry. An attractive solution is exploiting the regenerative potential of Mesenchymal Stem Cells (MSCs) isolated from adult tissues that could differentiate into osteoblasts and chondrocytes [1–3]. In this context, interest recently arose for MSCs from the lamina propria of the gingiva (GMSCs), that represents an easily accessible source from which MSCs can be isolated with minimally invasive techniques [4–6]. GMSCs can be propagated in vitro for long-time while maintaining a stable phenotype and can be induced to differentiate into the osteogenic lineage employing a variety of substances, including herbal-derived polyphenols [7–11].

Recently, interest arose for the potential of resveratrol (RV, trans 3,5,4′ trihydroxy-stylbene), a naturally occurring polyphenol, to prevent and cure bone loss-related diseases [12, 13]. RV shows anti-inflammatory [14] and anti-osteoclastic activities [15, 16] while showing osteoblastic differentiation promoting activities on MSCs [17–21]. However, the osteogenic response to RV has not been tested yet in human GMSCs (HGMSCs).

Stem cell differentiation implies a morpho-functional remodeling of the cell that is accomplished through dynamic and coordinated processes of macromolecular degradation and synthesis along with transcriptional and epigenetic reprogramming [22–24]. Macromolecular degradation in stem cells undergoing differentiation occurs via macro-autophagy (now on simply autophagy), which consists in the entrapment of cellular components such as organelles, membranes and cytosolic proteins within a double-membrane vesicle (the autophagosome) that will eventually fuse with lysosomes to form an autolysosome wherein the substrates will be degraded to completion [24, 25]. Autophagy is a stress-response and homeostatic process that plays a pivotal role in bone homeostasis [26]. However, whether and how autophagy is implicated in the osteogenic differentiation of MSCs remains to be elucidated yet. Here, we have investigated the functional role and the regulation of autophagy during the osteogenic differentiation of HGMSCs using RV as an inducer of autophagy [27] and of osteogenic differentiation of MSCs [18] at the same time. We show that RV synergizes with osteogenic inductive factors to accelerate the osteogenic differentiation of HGMSCs and that this effect is strictly dependent on the modulation of autophagy.

## Methods

### Isolation of human gingival mesenchymal stem cells

Human Gingival Mesenchymal Stem Cells (HGMSCs) were isolated from gingival tissue samples of adult healthy patients undergoing orthodontic surgery procedures. Each subject gave written informed consent, in accordance with the Helsinki Declaration, before their inclusion in the study. The Ethical Committee of Padova Hospital (Padova, Italy) approved the research protocol. After collection, gingival biopsies were briefly washed with Phosphate Buffered Saline (PBS; EuroClone, Milan, Italy), minced, then enzymatically digested with a solution of 3 mg/mL collagenase type I (Sigma-Aldrich, Saint Louis, MO, USA) and 4 mg/mL dispase (Sigma-Aldrich) in PBS for 2 h at 37 °C, as described elsewhere [28]. Once digested, the solution was filtered through 70 mm Falcon strainers (Becton & Dickinson, Franklin Lakes, NJ). The isolated cells were then cultured with Dulbecco’s Modified Eagle’s Medium (DMEM) high glucose (EuroClone), supplemented with 10% Fetal Bovine Serum (FBS; EuroClone), and 1% penicillin/streptomycin (P/S; EuroClone). Culture medium was refreshed twice a week. At 80–90% confluence, cells were detached with trypsin-EDTA solution (Sigma-Aldrich) and passaged repeatedly.

### Characterization of HGMSCs by flow cytometry

Adherent cells at passage 3 were dissociated and resuspended in flow cytometry staining buffer (R&D Systems, Minneapolis, MN, USA) at a final cell concentration of 1 × 10^6^ cells/mL. For surface markers characterization, the following fluorescent monoclonal mouse anti-human antibodies were used: CD73 APC (eBioscience™, Thermo Fisher Scientific, San Diego, CA, USA), CD90 BV510 (BD Biosciences, San Jose, CA, USA), CD105 PE-Cyanine7 (eBioscience™), CD14 PE (eBioscience™), CD34 APC-eFluor 780 (eBioscienceTM), and CD45 Pacific Orange (Thermo Fisher Scientific), as published elsewhere [29]. Cells were washed twice with 2 mL of flow cytometry staining buffer and resuspended in 500 μL of flow cytometry staining buffer. Fluorescence was evaluated by flow cytometry in Attune NxT flow cytometer (Thermo Fisher Scientific). Data were analyzed using Attune NxT software (Thermo Fisher Scientific).

### Cell culture and reagents

HGMSCs were cultivated under standard conditions (37 °C, 95% air: 5% CO_2_ v/v) in α-Minimum Essential Medium Eagle (α-MEM, Cod. M8042, Sigma-Aldrich, St. Luis, MO, USA) supplemented with 10% heat-inactivated Fetal Bovine Serum (FBS, cod. ECS0180L; Euroclone S.p.A., Milan, Italy), 2 mM L-glutamine (cod. G7513, Sigma-Aldrich) and 1% w/v of Penicillin/Streptomycin (cod. P0781, Sigma-Aldrich). For osteogenic differentiation, cells were incubated up to 21 days in α-MEM supplemented with 50 μg/mL of L-ascorbic acid 2-phosphate (Cod. 49,752, Sigma-Aldrich), 100 nM of dexamethasone (Cod. D1756, Sigma-Aldrich), and 10 mM of β-glycerophosphate (Cod. G9422, Sigma-Aldrich) (referred to as ‘differentiation medium’) [30]. Differentiation medium was replaced twice or thrice a week by adding all previous reagents. Resveratrol (RV, Cod. R5010, Sigma-Aldrich) was added to the standard or differentiation medium as indicated. Where reported, 5 μM spautin-1 (Sp1, Cod. SML0440, Sigma-Aldrich) was added to the culture medium.

HGMSCs were seeded on 35 mm Petri dishes at 80.000 cells per dish or on sterile glass coverslips at 10.000 cells per dish for western blot and immunofluorescence analysis, respectively. For histochemical staining with Alizarin Red Staining, the cells were cultured on 24-well plates at 20.000 cells per well and let adhere 24 h before treatments.

### Antibodies

The following primary antibodies were employed for western blotting and immunofluorescence techniques: rabbit monoclonal anti-RUNX2 (Cod. 12,556, Cell Signaling Technology Inc., Danvers, MA, USA), rabbit polyclonal anti-collagen Type 1 alpha 1 (Cod. NB600–408, Novus Biological Centennial, USA), mouse monoclonal anti-osteopontin (Cod. MA5–17180, Thermo Fisher Scientific Inc., Waltham, MA, USA), mouse monoclonal anti-osteocalcin (Cod. sc-74,495, Santa Cruz Biotechnology Inc., Dallas, TX, USA), mouse monoclonal anti-beclin-1 (Cod. 612,112, BD Biosciences, San Jose, CA, USA), rabbit monoclonal anti-phospho-beclin-1 (Ser93) (Cod. 14,717, Cell Signaling Technology Inc.), rabbit monoclonal anti-PI3 Kinase Class III (Vps34, Cod. 4263, Cell Signaling Technology Inc.), rabbit polyclonal anti-LC3B (Cod. L7543, Sigma-Aldrich), mouse monoclonal anti-β-tubulin (Cod. T5293, Sigma-Aldrich Corp.), mouse monoclonal anti- β-actin (Cod. A5441, Sigma-Aldrich), mouse monoclonal anti-LAMP1 (Cod. 555,798, Becton, Dickinson and Company, New Jersey, NJ, USA), rabbit polyclonal anti-AMPKα (Cod. 2532, Cell Signaling Technology Inc.) and rabbit monoclonal anti-phospho-AMPKα (Thr172) (Cod. 2535, Cell Signaling Technology Inc.).

### Western blotting

HGMSCs were homogenized in RIPA buffer (0.5% deoxycholate, 1% NP-40, 0.1% Sodium Dodecyl Sulfate in PBS solution) supplemented with protease inhibitor cocktail and phosphatase inhibitors (Na_3_VO_4_ and NaF). Proteins were determined by Bradford assay, denatured with 5X Loading buffer at 95 °C for 10 min and fractionated by SDS-PAGE at different acrylamide percentage (15, 12.5, 8% or 6%) according to the m.w. of the target protein. Molecular weight markers were PageRuler Prestained Protein Ladder (Cod. 26,616, Thermo Fisher Scientific Inc.) for 15 and 12.5% gels, and Spectra Multicolor High Range Protein Ladder (cod. 26,625, Thermo Fisher Scientific Inc.) for 8 and 6% gels. After PAGE, the proteins were blotted onto PVDF membranes (cod. 162–0177, Bio-Rad, Hercules, CA, USA). Membranes were blocked with 5% non-fat milk (cod. 68,514–61-4, SERVA Electrophoresis GmbH, Heidelberg, Germany) containing 0.2% Tween-20 for 1 h at room temperature (RT), incubated with specific primary antibody overnight at 4 °C and, thereafter, with the secondary HRP-conjugated antibody for at least 1 h at RT. β-tubulin and β-actin were used as homogenate protein loading control. Membranes were developed with the enhanced chemiluminescence method (ECL, cod. NEL103E001 EA; PerkinElmer Inc., Waltham, MA, USA). The relative band intensity was acquired with the VersaDOC Imaging System apparatus (Bio-Rad) and quantified by Quantity One 4.5.0 software (Bio-Rad). At least three independent replicates per each western blot were performed.

### Immunofluorescence

HGMSCs plated on sterile coverslips were incubated as indicated. At the end, the cells were washed with PBS, fixed with ice-cold 100% methanol and permeabilized with 0.2% Triton X-100 in PBS for 10 min. Then, cells were incubated with the specific primary antibodies overnight at 4 °C. The following day, the coverslips were washed with 0.1% Triton X-100 in PBS and incubated for 1 h at RT with goat-anti-rabbit IgG Alexa Fluor™ plus 488 (Cod. A32731, Thermo Fisher Scientific Inc.) or goat-anti-mouse IgG Alexa Fluor™ plus 555 (Cod. A32727, Thermo Fisher Scientific Inc.) secondary antibodies, as appropriate. Nuclei were stained with DAPI (4′,6-diamidino-2-phenylindole, cod. 32,670, Sigma-Aldrich Corp.). Thereafter, coverslips were mounted onto glasses using slow-FADE anti-FADe reagent (Cod. S36936, Life Technologies Ltd) and fluorescence images were acquired with the Leica DMI6000 fluorescence microscope (Leica Microsystems AG, Wetzlad, DE). The fluorescence intensity was measured by Image-J 1.48v software (http://imagej.nih.gov/ij/) and indicated as IntDen, for either single channel and co-labelling (red + green = yellow). IntDen (Integrated Density) refers to the average value of fluorescence in a selected area normalized to the number of cells. At least three slides were prepared for each experimental condition and fluorescence in up to 100–200 cells in total present in six to ten microscopic fields randomly chosen was quantified. Images shown are representative of at least three separate experiments.

### Assessment of osteogenic differentiation

The presence of calcium deposition in the extracellular matrix, a sign of osteoblastic activity, was detected by Alizarin Red S staining [31]. At the end of incubation in medium containing or not the osteogenic differentiation factors and/or resveratrol, the cell culture was washed with PBS, fixed in 10% formaldehyde for 30 min at room temperature (RT), rinsed twice with deionized water and stained with 40 mM Alizarin Red S (Cod. TMS-008-C, Sigma-Aldrich), pH 4.1, for 45 min at RT. Then, the cultures were washed four times with deionized water to remove non-specifically bound stain. After drying, stained monolayers were observed and imaged under the phase microscope. The area of calcium deposits, indicated as Calcium Deposition (% Area), was calculated using the Image-J 1.48v software.

### Statistics

All experiments were performed at least three times, separately. Data in histograms are shown as average ± S.D. GraphPad Prism was employed (GraphPad Software Inc.) for statistical analysis. Statistical significance of the data was given by one-way ANOVA analysis of variance followed by Tukey’s test. Differences were considered significant for *p < 0.05; **p < 0.01; ***p < 0.001.

## Results

### Characterization of human gingival mesenchymal stem cells

**Fig. 1:**
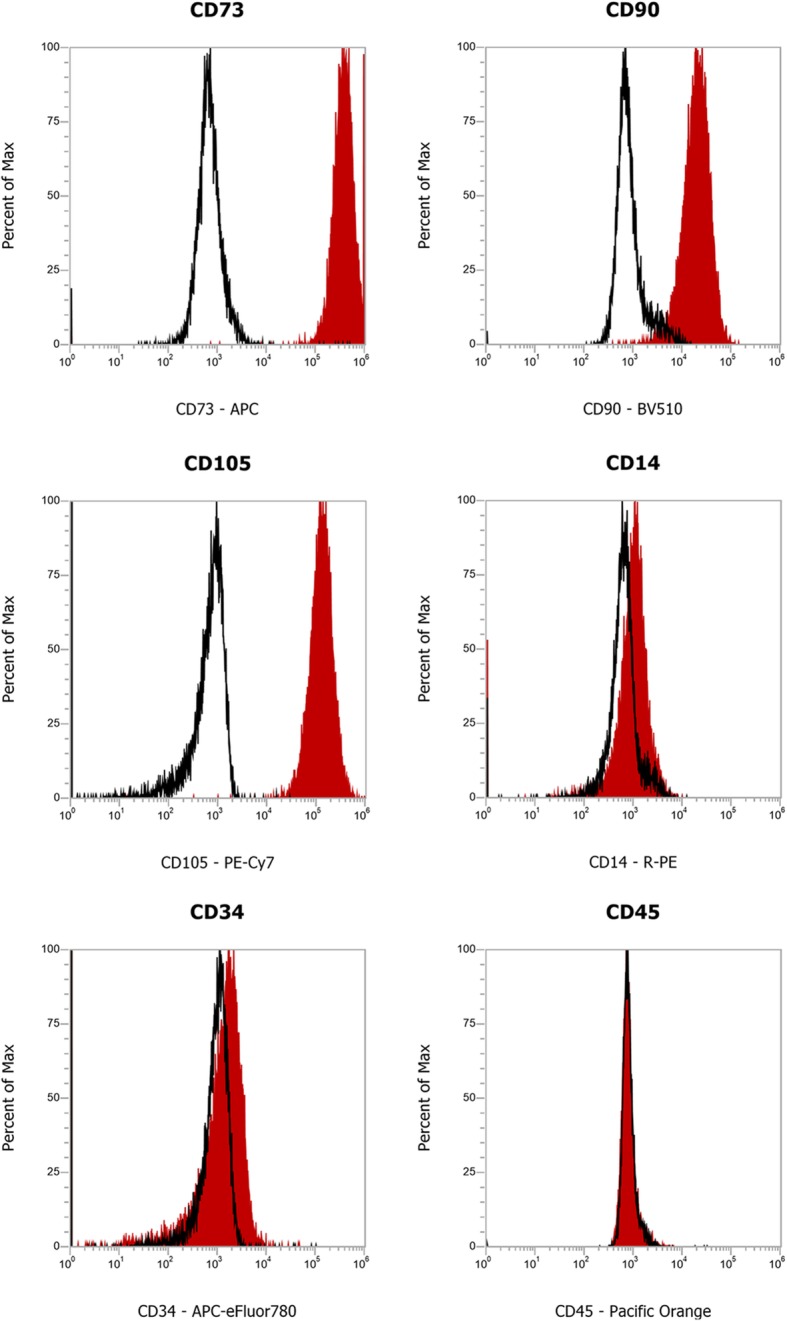
Isolation and characterization of Human Gingival Mesenchymal Stem Cells. Characterization of cell surface markers in HGMSCs at passage 3 by flow cytometry. The stem cells isolated from the gingival biopsies are positive to CD73, CD90, and CD105 MSCs-specific markers, and negative to CD14, CD34, and CD45 hematopoietic markers

The HGMSCs isolated from the gingival samples were characterized according to their surface protein expression by flow cytometry. As shown in Fig. 1, the cells were found positive for the established MSCs-specific surface markers CD73, CD90, and CD105 [32]. Flow cytometry immunophenotyping also revealed the negativity to CD14, CD34, and CD45, confirming the absence of hematopoietic cells in the isolated stem cells population.

### Resveratrol promotes the osteogenic differentiation of human gingival mesenchymal stem cells

**Fig. 2:**
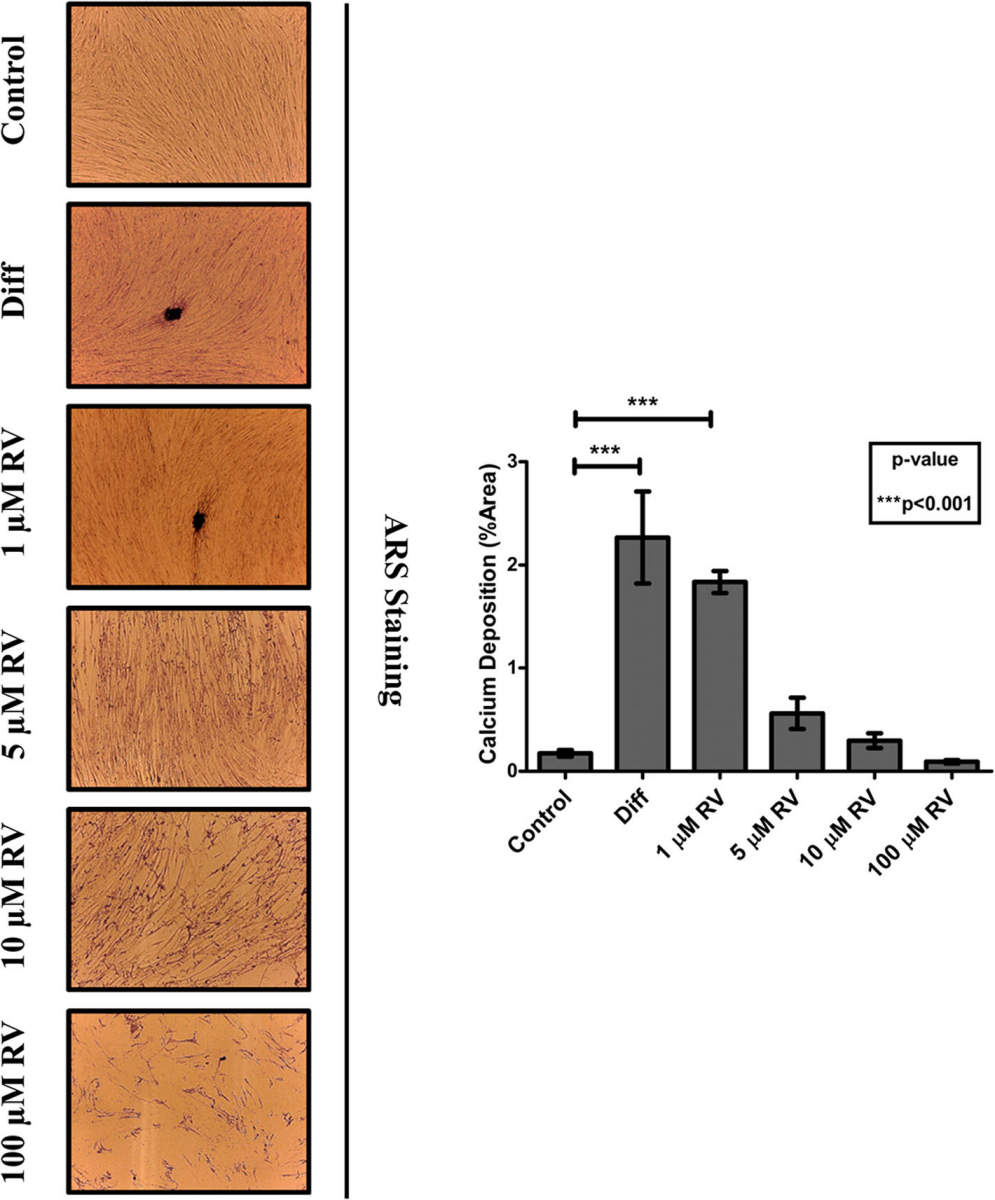
Resveratrol promotes the osteoblastic differentiation of Human Gingival Mesenchymal Stem Cells. Adherent HGMSCs were cultured for 21 days in control medium supplemented or not with resveratrol (RV) at the indicated concentration or in differentiation medium (Diff) containing the three osteoblastic inductive factors dexamethasone, β- glycerophosphate and ascorbic acid. At the end, the cultures were processed for Alizarin Red S staining of extracellular calcium deposits. The stained area was quantified using the ImageJ software

**Fig. 3:**
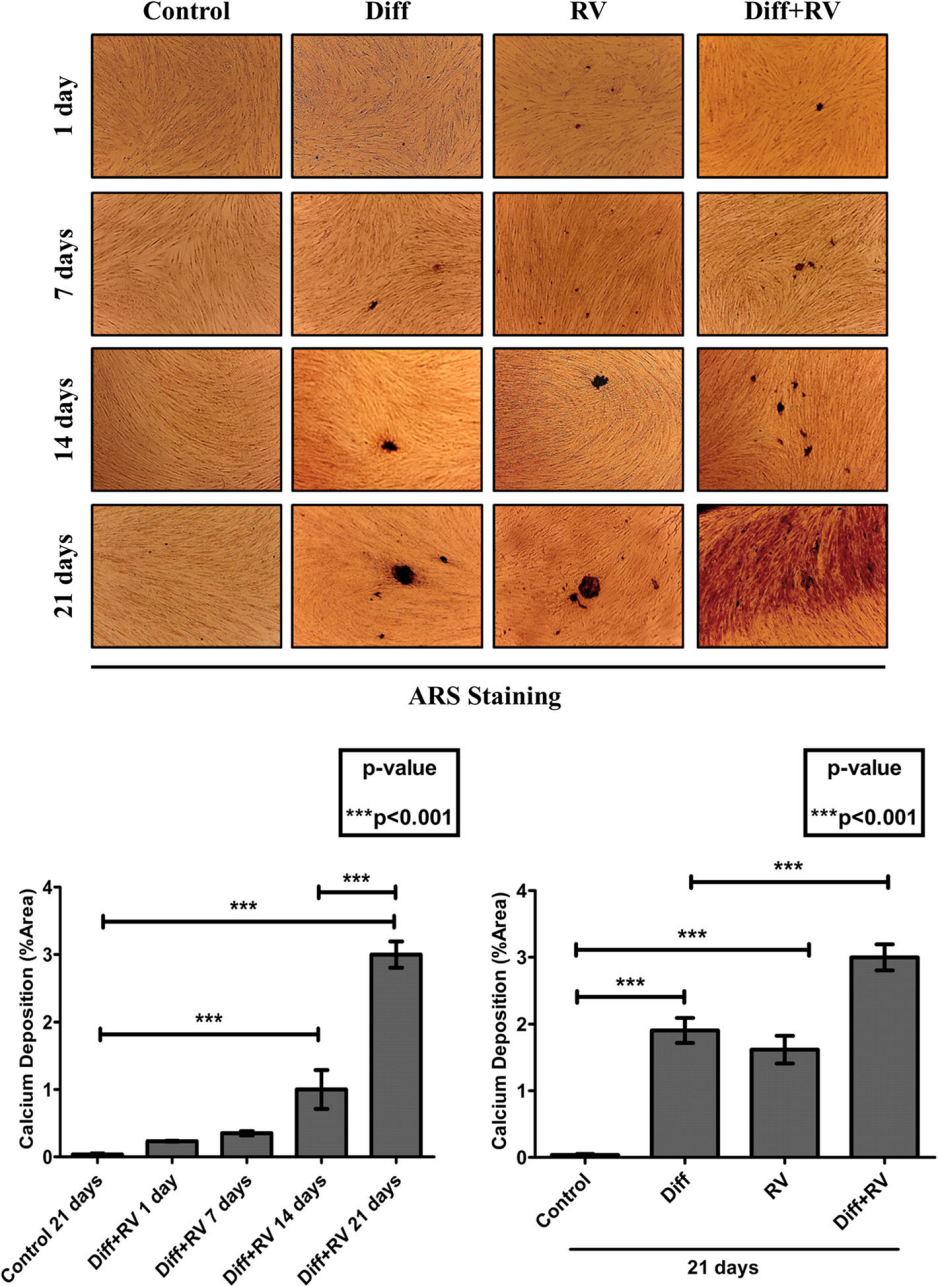
Resveratrol synergizes with osteogenic inductive factors to accelerate osteoblastic differentiation of Human Gingival Mesenchymal Stem Cells. Adherent HGMSCs were cultured for 1 to 21 days in control medium or in differentiation medium (Diff) supplemented or not with 1 μM resveratrol (RV). At the end, the cultures were processed for Alizarin Red S staining of extracellular calcium deposits. Quantification of stained area in the time-course is reported in the histogram

**Fig. 4:**
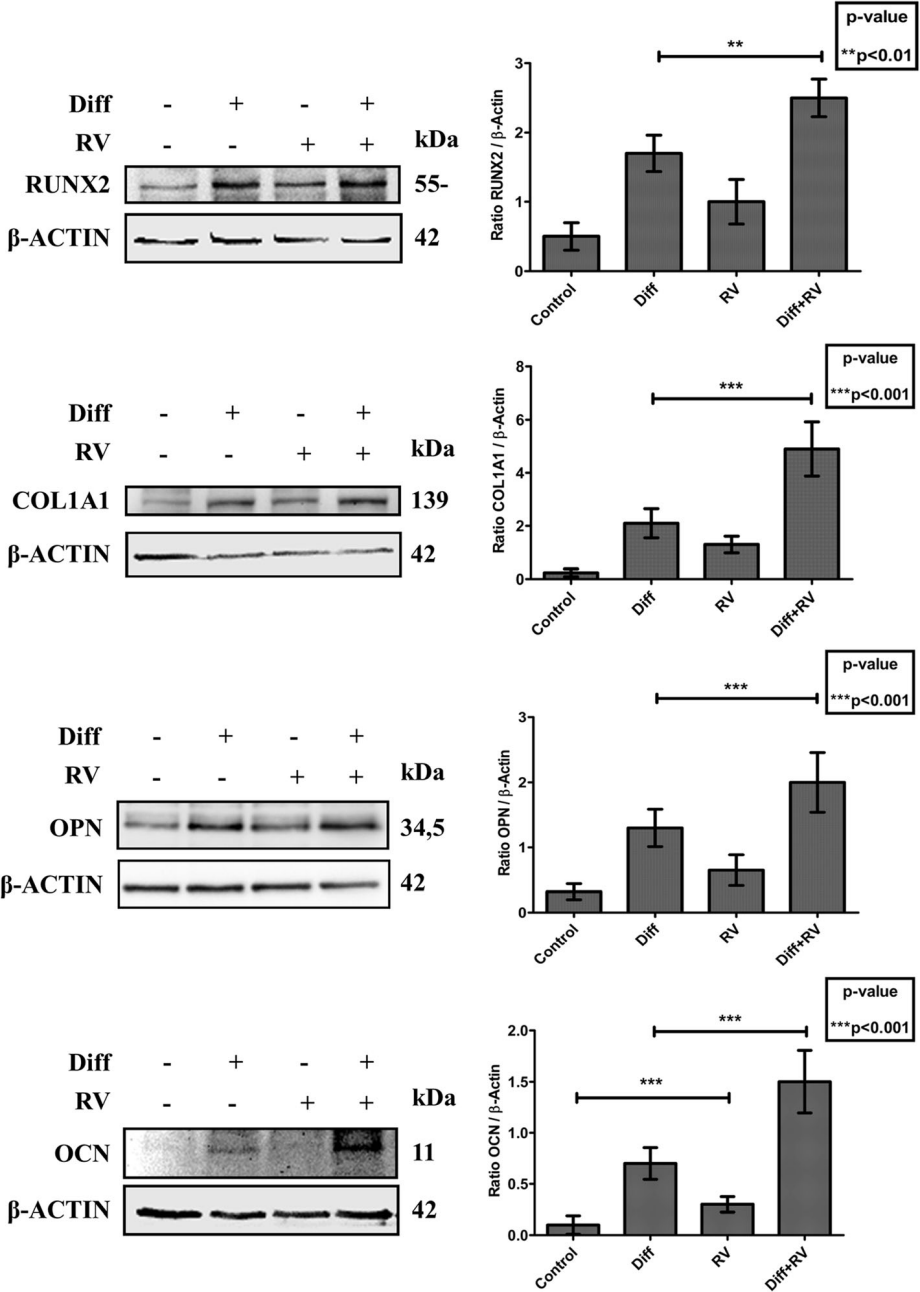
Expression of osteoblastic differentiation markers in Human Gingival Mesenchymal Stem Cells. Adherent HGMSCs were cultured for 21 days in control medium or in differentiation medium (Diff) supplemented or not with 1 μM resveratrol (RV). At the end, cell homogenates were processed for western blotting analysis of the expression of the osteoblastic transcription factor RUNX-2 and of the osteogenic differentiation markers COL1A1, OPN and OCN. Densitometry of the specific bands (average ± S.D.) of three independent experiments is shown in the histograms

**Fig. 5:**
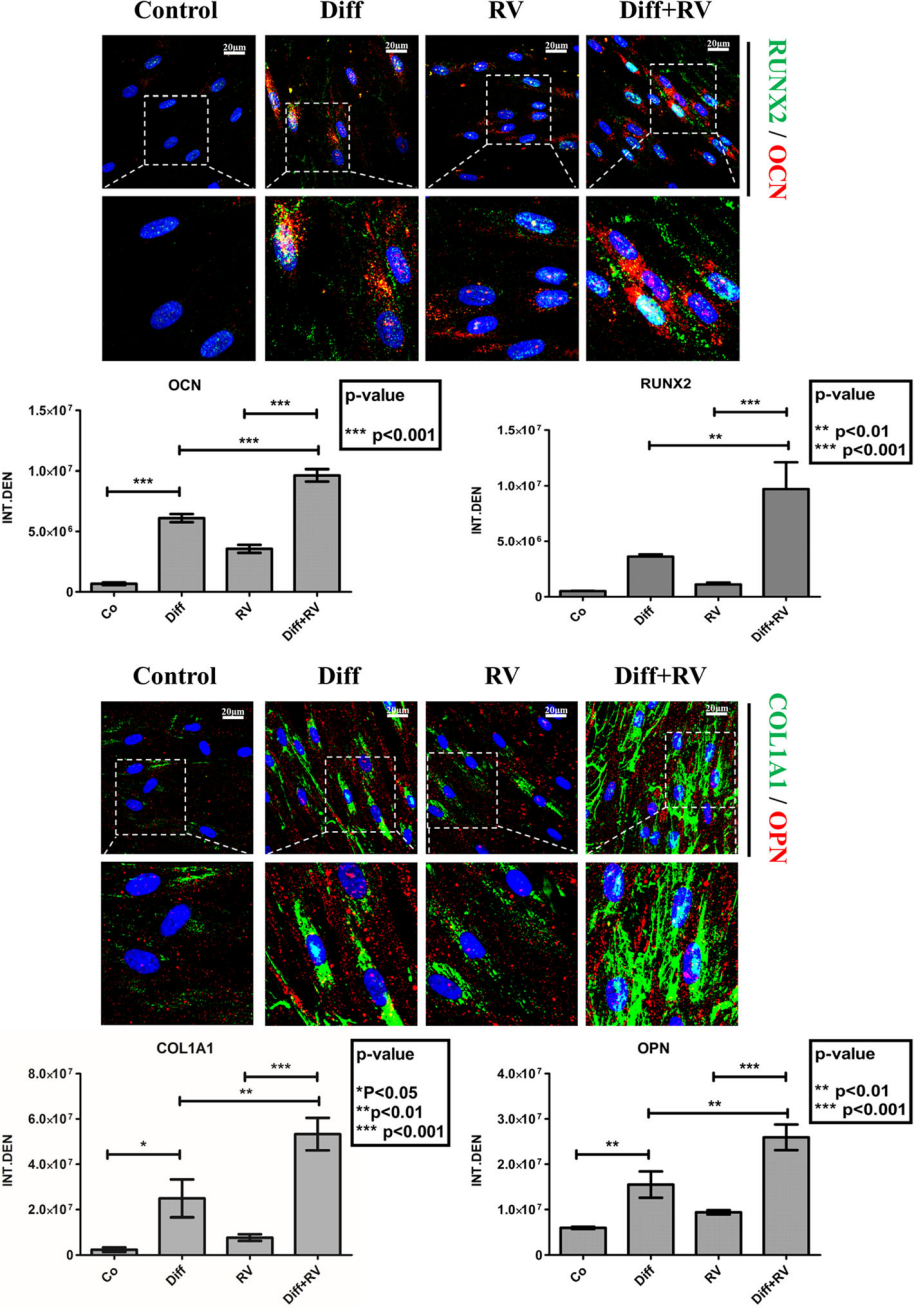
Immunofluorescence staining of osteoblastic differentiation markers in Human Gingival Mesenchymal Stem Cells. HGMSCs were plated on sterile coverslips, let adhere and cultured for 21 days in control medium or in differentiation medium (Diff) supplemented or not with 1 μM resveratrol (RV). At the end, the coverslips were fixed and processed for immunofluorescence staining of the osteogenic differentiation markers. Fluorescence staining was quantified with the ImageJ software

To determine the optimal conditions for osteogenic differentiation by RV, HGMSCs were incubated for up to 21 days with RV in concentration ranging from 1 to 100 μM. As positive control, the cells were incubated in a medium supplemented with the osteogenic differentiation factors dexamethasone, β-glycerophosphate and ascorbic acid (from now on, referred as ‘differentiation medium’). To monitor the mineralization associated with osteogenic differentiation of HGMSCs the cultures were stained with Alizarin Red S to detect the calcium deposits in the extracellular space. The mineralization was clearly detectable after a minimum of 7–14 days culture in differentiation medium (not shown). Representative images in Fig. 2 (and relative quantification) show that the osteogenic differentiation promoted by RV is maximal at 1 μM and declines when concentration raises up to 100 μM, which turned out to be toxic. When 1 μM RV was added to the differentiation medium, osteogenic differentiation of HGMSCs (as mirrored by the mineralization of the extracellular matrix) was accelerated, indicating a synergism between RV and osteogenic differentiation factors (Fig. 3). To characterize at molecular level this effect, we analyzed the expression of signaling and structural protein markers of the osteogenic differentiation [33]. Western blotting showed that RUNX2, the transcription factor of osteocalcin (OCN) and of other genes associated with osteoblast differentiation, [34, 35] was upregulated in the HGMSCs cultured in the differentiation medium or in the presence of RV alone (though to a lower extent in the latter case) (Fig. 4). Interestingly, when RV was added to the differentiation medium, the expression of RUNX2 was further induced compared to the culture conditions in either the differentiation medium or RV alone (Fig. 4). The combined expression of collagen 1 (COL1A1) and of osteopontin (OPN) is suggestive of differentiation of MSCs toward the osteogenic line, while OCN synthesis is switched on in osteoblasts and its expression is therefore proofing that osteoblast differentiation indeed occurred [33]. Compared to the osteogenic inductive factors, RV alone elicited a slight increase in the expression of these markers (Fig. 4). However, when RV was added to the osteogenic differentiation medium it greatly stimulated the expression of these three proteins in a synergistic manner with the osteogenic factors (Fig. 4). To prove further the acquisition of an osteogenic phenotype by HGMSCs under these culture conditions, we performed the immunofluorescence co-staining of the above markers. Representative images and their quantification are shown in Fig. 5. The data confirm that after 21 days of treatment with 1 μM RV alone the expression of these markers was weakly induced, while the addition of RV to the osteogenic differentiation medium synergistically augmented their expression in the cells. Noteworthy, RV greatly stimulated the nuclear translocation of RUNX2 (Fig. 5a).

### Osteogenic differentiation of human gingival mesenchymal stem cells associates with induction of autophagy

**Fig. 6:**
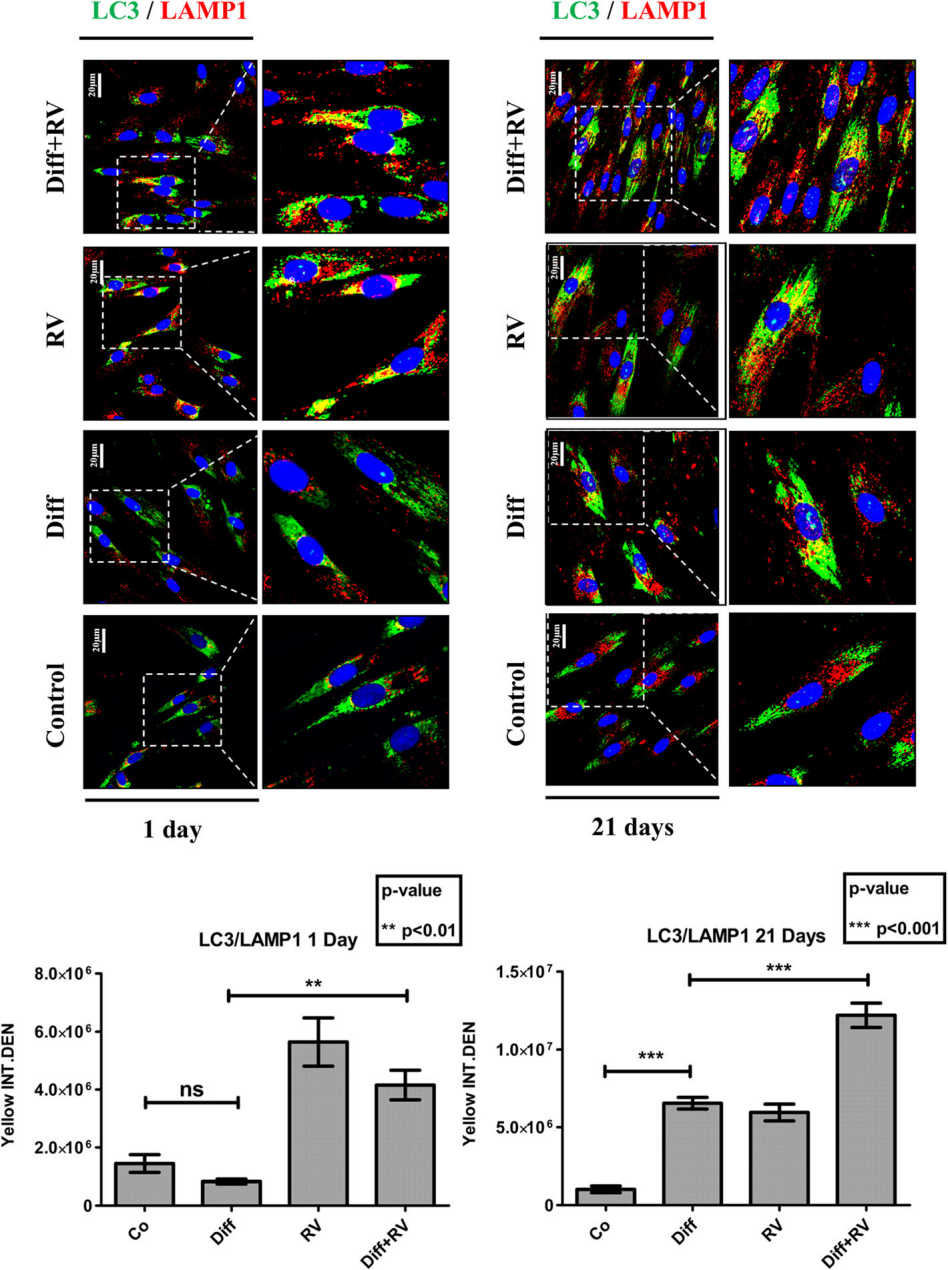
Osteoblastic differentiation of Human Gingival Mesenchymal Stem Cells associates with induction of autophagy. HGMSCs were plated on sterile coverslips, let adhere and cultured for 1 to 21 days in control medium or in differentiation medium (Diff) supplemented or not with 1 μM resveratrol (RV). At the end, the coverslips were fixed and processed for immunofluorescence staining of the autophagy markers LC3 (marker of autophagosomes) and LAMP1 (marker of lysosomes). Fluorescence staining was quantified with the ImageJ software. Integrated fluorescence intensity of co-labeled area (yellow) was calculated and reported in histograms

**Fig. 7:**
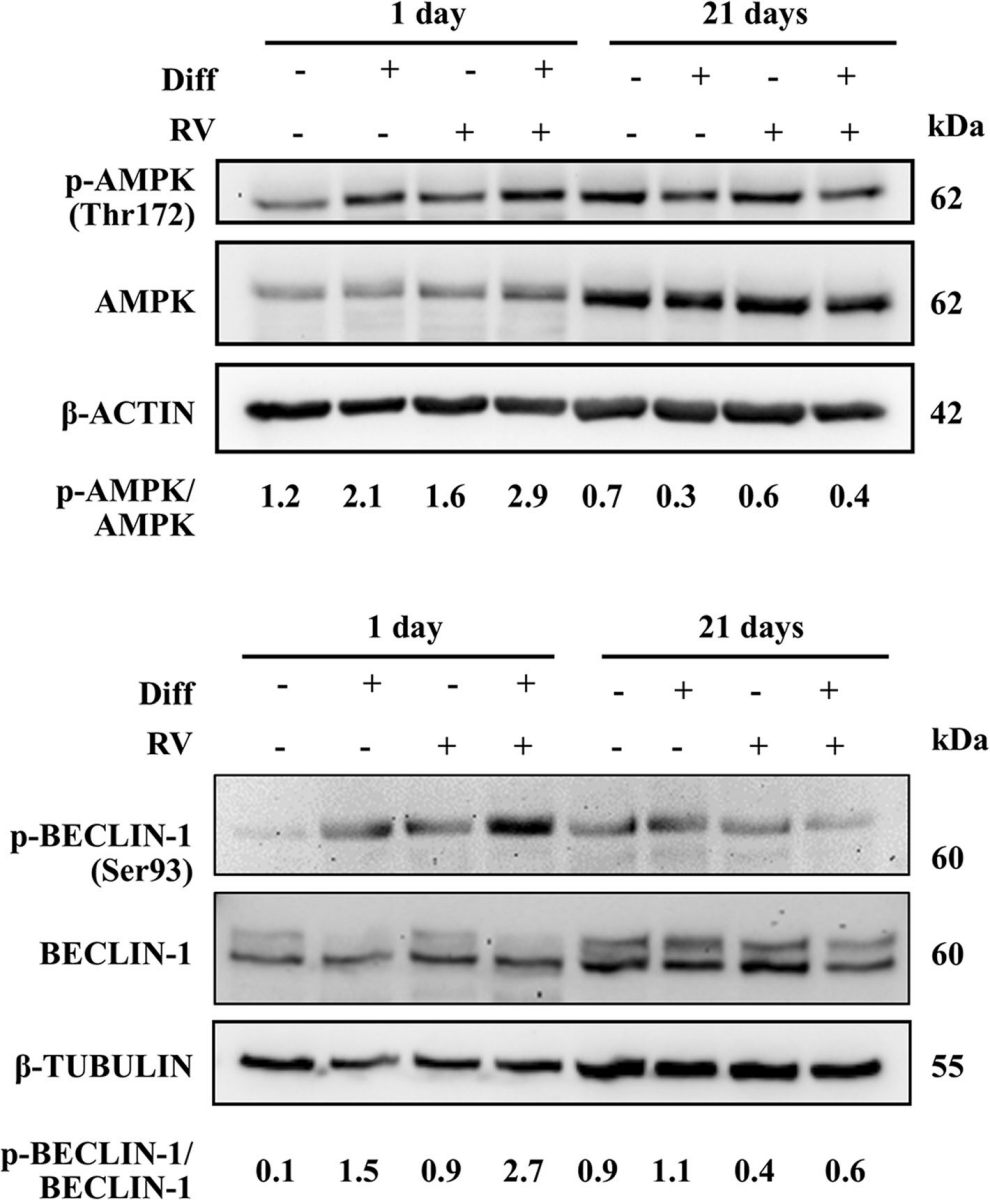
Osteoblastic differentiation of Human Gingival Mesenchymal Stem Cells associates with modulation of the AMPK-BECLIN-1 autophagy signaling pathway. HGMSCs were cultured for 1 to 21 days in control medium or in differentiation medium (Diff) supplemented or not with 1 μM resveratrol (RV). At the end, cell homogenates were processed for western blotting analysis of the expression of activated (phosphorylated) AMPK and BECLIN-1, two signaling proteins that govern autophagy. Densitometry (arbitrary units) is included. Similar data were reproduced in another independent experiment

To see if autophagy is involved in the osteogenic differentiation of HGMSCs we first performed the immunofluorescence staining of autophagic vacuoles with antibodies specific to LC3, a lipidated protein specifically associated with the membranes of the autophagosomes, and to LAMP1, an integral protein of the lysosomal membranes [36]. The co-labeling marks the autolysosome and is indicative of the effective fusion of autophagosomes with lysosomes. Representative images taken at day 1 and day 21 are shown in Fig. 6. ImageJ quantification of co-labeled vesicles (autolysosomes) indicated that the autophagy flux was greatly and promptly (since day 1) stimulated by RV, while it was initially (at day 1) downregulated and later (at day 21) induced by the osteogenic differentiation factors. Remarkably, the formation and accumulation of autolysosomes were greatly stimulated in the cells cultured in differentiation medium supplemented with RV (Fig. 6). As a further proof of the induction of autophagy during HGMSCs differentiation, we analyzed the AMPK-BECLIN-1 pathway, as primary candidate of the signaling pathway triggered by RV [37]. It was found that under osteogenic inductive culture conditions AMPK was active and, particularly, BECLIN-1 was synergistically activated by the combination of RV and osteogenic differentiation factors (Fig. 7). It is to be noted that by day 21, when the culture reached confluency and osteoblastic differentiation almost reached completion, the AMPK-BECLIN-1 pathway was switched off (Fig. 7).

### Inhibition of BECLIN-1-depedent autophagy impairs osteogenic differentiation of HGMSCs by resveratrol

**Fig. 8:**
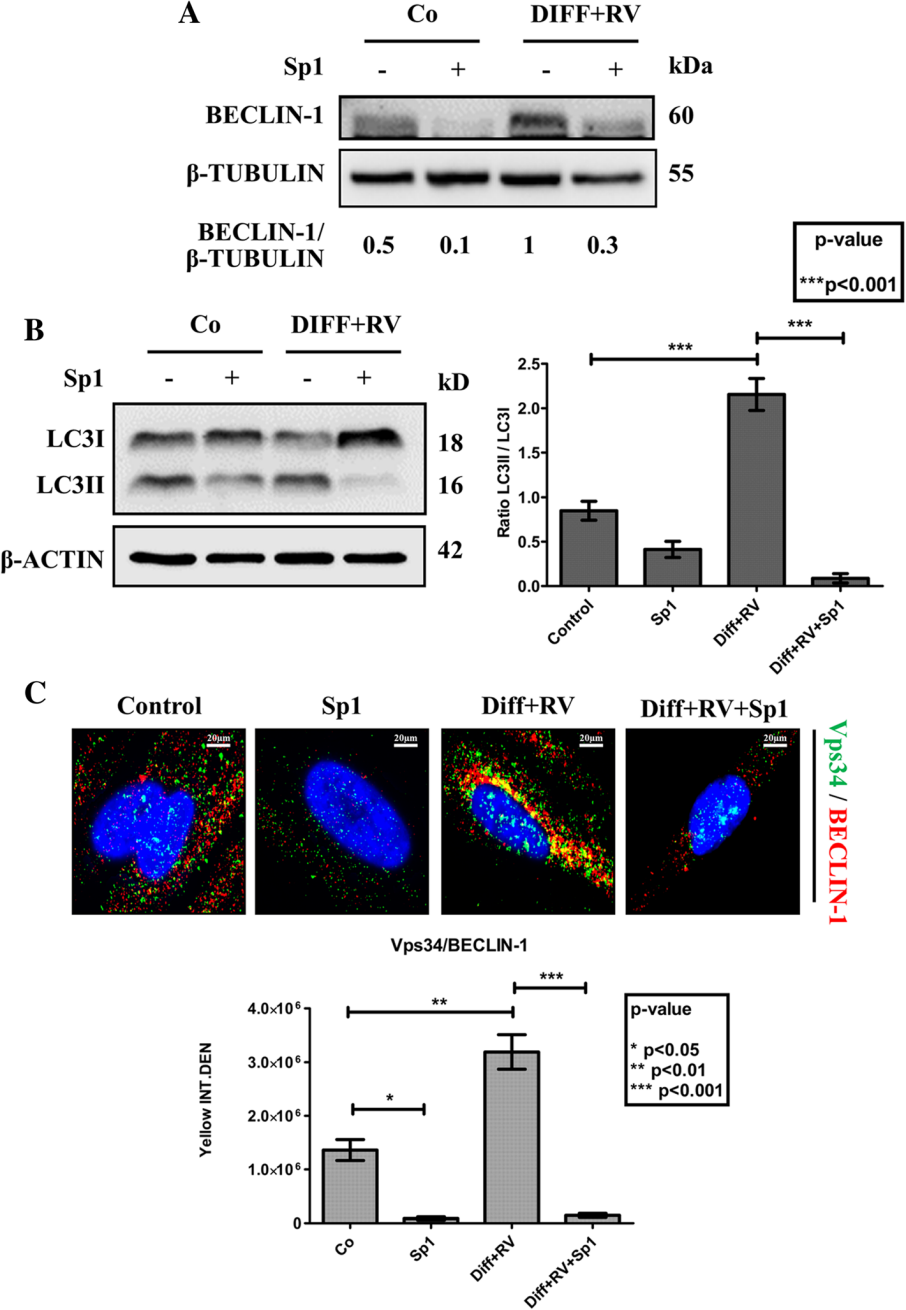
Spautin-1 abrogates induction of BECLIN-1-dependent autophagy in Human Gingival Mesenchymal Stem Cells cultivated in osteoblastic differentiation condition. HGMSCs were cultured for 21 days in control medium or in differentiation medium supplemented with 1 μM resveratrol (Diff + RV) in the absence or in the presence of spautin-1 (Sp1). At the end, cell homogenates were processed for western blotting analysis of the expression of **a** BECLIN-1 (target of spautin-1) and of **b** LC3 (marker of autophagosome). Densitometry (arbitrary units) of the specific bands is included. Data were reproduced in three independent experiments. The ratio LC3-II/LC3-I is assumed as an index of autophagosome and autolysosome accumulation in the cell. **c** HGMSCs were plated on sterile coverslips, let adhere and cultured for 21 days in control medium or in differentiation medium supplemented with 1 μM resveratrol (Diff + RV) in the absence or the presence of spautin-1 (Sp1). At the end, the coverslips were fixed and processed for immunofluorescence staining of the autophagy interactome markers Vps34 (PI3KC3) and BECLIN-1. Fluorescence staining was quantified with the ImageJ software. Integrated fluorescence intensity of co-labeled area (yellow) was calculated and reported in histograms. Data from three coverslips per condition reproduced in three separate experiments

**Fig. 9:**
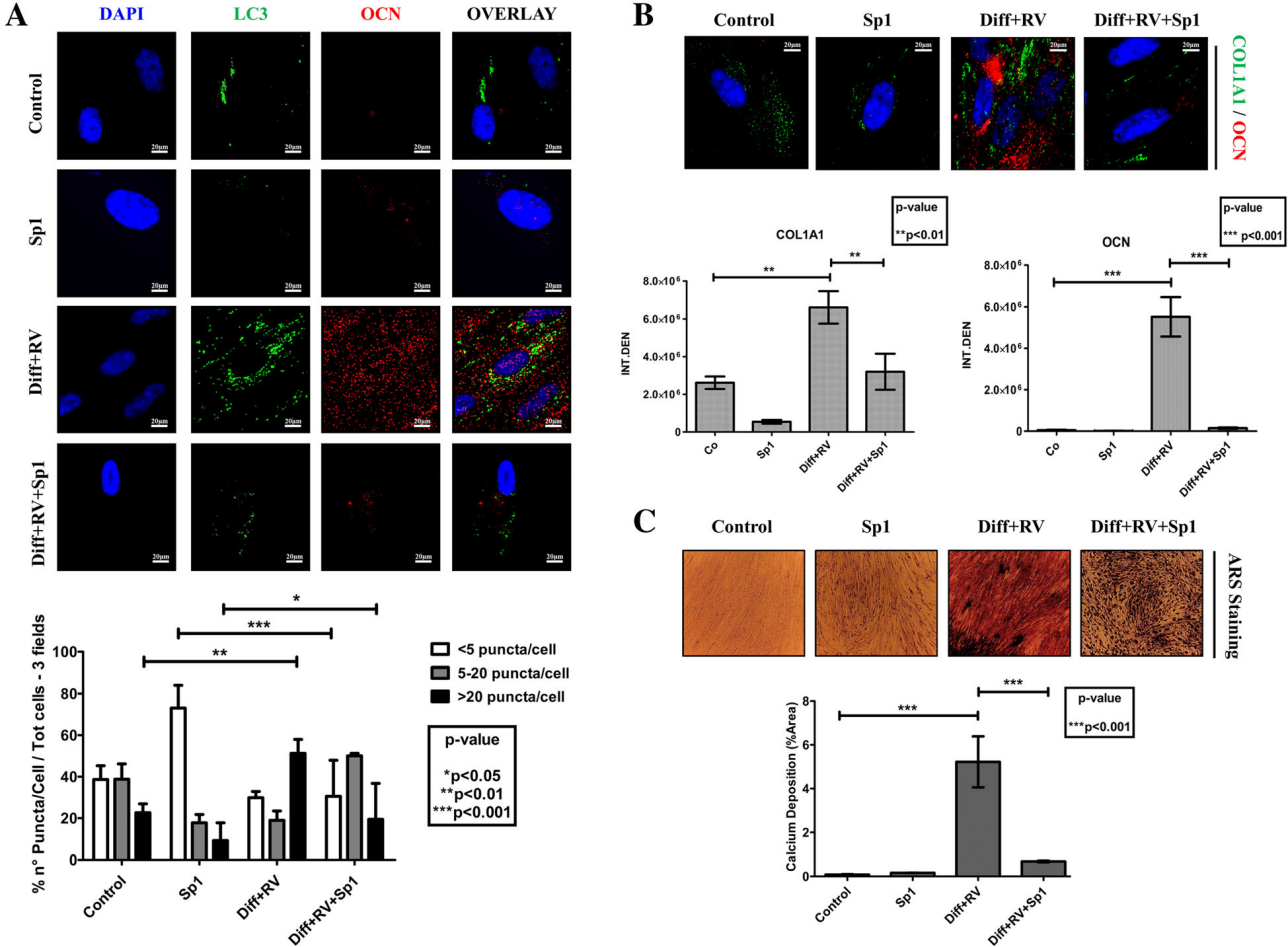
Spautin-1 prevents the autophagy-associated osteoblastic differentiation of Human Gingival Mesenchymal Stem cells. **a** HGMSCs were plated on sterile coverslips, let adhere and cultured for 21 days in control medium or in differentiation medium supplemented with 1 μM resveratrol (Diff + RV) in the absence or the presence of spautin-1 (Sp1). The coverslips were then fixed and processed for immunofluorescence staining of the autophagy marker LC3 and of the osteoblastic differentiation marker OCN. As an index of autophagy in the cells, LC3 puncta were quantified (as per the guidelines 36). **b** HGMSCs were plated and treated as in **a** and the coverslips processed for immunofluorescence staining of the osteoblastic differentiation markers OCN and COL1A1. Integrated fluorescence intensity quantified with the ImageJ software is shown in the histograms. **c** HGMSCs cells were plated on plastic and treated as in **a** and at the end the cultures were processed for Alizarin Red S staining of extracellular calcium deposits. The stained area was quantified using the ImageJ software

**Fig. 10:**
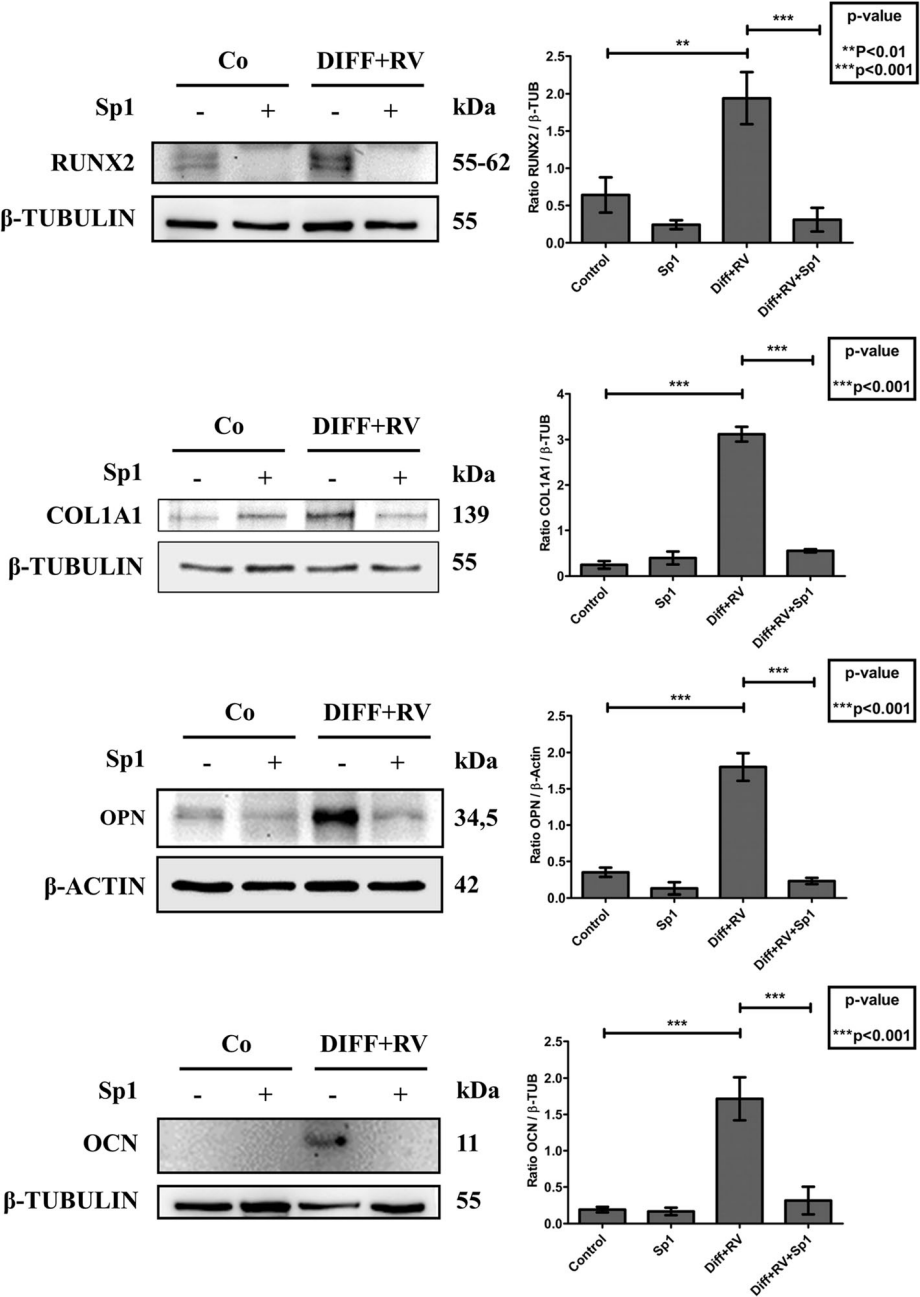
Spautin-1 prevents the expression of osteoblastic differentiation markers in Human Gingival Mesenchymal Stem cells. HGMSCs were cultured for 21 days in control medium or in differentiation medium supplemented with 1 μM resveratrol (Diff + RV) in the absence or in the presence of spautin-1 (Sp1). At the end, cell homogenates were processed for western blotting analysis of the expression of the osteoblastic transcription factor RUNX-2 and of the osteogenic differentiation markers COL1A1, OPN and OCN. Densitometry of the specific bands (average ± S.D.) of three independent experiments is shown in the histograms

Finally, we investigated whether autophagy was actively involved in the differentiation process or it was just an accompanying epiphenomenon. To this end, we monitored the occurrence of osteogenic differentiation in HGMSCs cultivated in the presence of spautin-1, a potent inhibitor of autophagy that promotes the proteasome-mediated degradation of BECLIN-1 [38]. We first determined the appropriate concentration of spautin-1 that could inhibit chronically (for 21 days) autophagy with no toxic side effect on HGMSCs cell viability (data not shown). 5 μM Spautin-1 effectively depleted the cell of BECLIN-1 (Fig. 8a), resulting in a strong inhibition of autophagy as shown by the impaired conversion of LC3-I into LC3-II (Fig. 8b). This was further confirmed by the lack of interaction between BECLIN-1 and Vps34 (aka PI3KC3) in the cells cultivated in RV-supplemented differentiation medium in the presence of spautin-1, as shown by immunofluorescence co-staining (Fig. 8c). Next, we searched for the mechanistic link between autophagy and osteogenic differentiation. The chronic incubation in differentiation medium supplemented with RV led to the synthesis and accumulation of OCN in the cells with upregulated autophagy, as indicated by the presence of LC3-positive dots (Fig. 9a). However, in the parallel cultures co-treated with spautin-1 the number of LC3-positive dots per cell was greatly reduced to the level in controls and the OCN staining was faintly visible (Fig. 9a). Similarly, intense fluorescent staining of OCN and COL1A1 was apparent in the cells cultivated in RV-supplemented differentiation medium, while it was no apparent in the parallel culture co-treated with Sp-1 (Fig. 9b). From a functional point of view, spautin-1 inhibition of autophagy resulted in impaired mineralization of the extracellular matrix, as indicated by the lack of Alizarin Red-positive deposits of calcium (Fig. 9c). Additionally, western blotting of RUNX2, COL1A1, OPN and OCN showed that the expression of these markers of osteogenic differentiation was completely prevented in the cultures exposed to Sp-1 despite the concomitant presence in the medium of RV and osteogenic inductive factors (Fig. 10). From these data we may conclude that autophagy is functionally linked to osteogenic differentiation of HGMSCs.

## Discussion

Autophagy is the major pathway for the cellular bulk degradation associated with the remodeling of cellular structures during differentiation of MSCs [23, 24]. During cellular differentiation, the autophagy pathway is finely tuned to meet the metabolic needs associated with the morpho-functional changes [22]. Two principal signaling pathways converging on the ULK1 complex control autophagy: the mTORC1 pathway, with inhibitory function, and the AMPK pathway, with activating function [39]. The mTORC1 pathway is positively triggered by growth factors and availability of nutrients, the AMPK pathway is activated when there is a lack of ATP production because of lack of nutrients or mitochondrial poisoning [40]. The AMPK-ULK1 pathway was shown to positively regulate autophagy-dependent mitochondrial homeostasis in embryonic stem cells, contributing to stemness properties [41]. Consistently, autophagy plays a role in maintaining the stemness properties and is modulated during stem cell differentiation [42]. In this work, we analyzed the contribution of autophagy in the osteoblastic differentiation of HGMSCs induced by RV or the osteoblastic inductive factors dexamethasone, β-glycerophosphate and ascorbic acid or their combination. We demonstrate that RV induces the osteoblastic differentiation of HGMSCs when used at 1 μM, while it is toxic at concentrations above 10 μM. Our results are in agreement with similar studies conducted on human bone-derived MSCs [17] and human embryonic stem cells [18]. Compared to the osteogenic inductive factors, RV elicited a less pronounced differentiation by day 21. It is likely that prolonging the incubation with RV would eventually attain a full osteoblastic differentiation. RV induced the expression and promoted the nuclear translocation of the osteogenic transcription factor RUNX2. Remarkably, RV synergized with the osteogenic inductive factors accelerating the osteoblastic differentiation of HGMSCs, as indicated by anticipation and increased mineralization of the extracellular matrix. Signs of osteoblastic differentiation such as OCN synthesis and extracellular calcium deposits became detectable after 7 days of culture. Osteoblastic differentiation of HGMSCs was strictly dependent on BECLIN-1 dependent autophagy, as demonstrated by the observation that it was prevented by spautin-1 induced depletion of BECLIN-1 and consequent inhibition of autophagy. It is likely that RV accelerated the osteoblastic differentiation of HGMSCs cultivated in the osteogenic differentiation medium because of its strong stimulation of autophagy. RV has been shown to induce autophagy in mouse embryonic stem cells via activation of the AMPK/ULK1 pathway, and this correlated with enhanced pluripotency of the cells [37]. RV mimics a situation of energy restriction and activates the AMPK pathway regardless of the presence of nutrients, leading to activation of autophagy [27]. Activation of AMPK bypasses the block by mTORC1 and triggers autophagy through direct activating phosphorylation of ULK1 and of BECLIN-1 [43, 44]. Under metabolic stress conditions, the parallel activation of mTOR and of AMPK allows the coordinated and contemporary protein degradation and protein synthesis processes, with the former providing the amino acids needed for the latter [45]. We observed that AMPK was transitorily activated in HGMSCs undergoing cell differentiation and it was down-regulated when osteoblastic differentiation was achieved. This same pattern was paralleled by phosphorylated Ser93 BECLIN-1, which is operated by AMPK [44]. Modulation of AMPK activation drives osteoblast differentiation: it is induced during early differentiation and its silencing or inhibition causes bone loss, yet its constitutive activation prevents full differentiation [46]. This modulation was paralleled by modulation of autophagy, suggesting that down-regulation of AMPK-dependent autophagy could favor glycolysis, which is necessary in the late stages of differentiation [46]. Interestingly, similar findings were reported in the myoblast to myotube differentiation of muscle satellite cells, which are regarded as stem-like cells [47]. Thus, down-regulation of the AMPK-BECLIN-1 pathway is consistent with the progressive downregulation of autophagy to basal levels once that full differentiation is achieved when it is no more requested the degradation of redundant or unwanted cell components.

## Conclusions

In summary, here, we provide for the first time the evidence that RV and osteogenic inductive factors synergize to induce the osteoblastic differentiation of HGMSCs and that this process relies on modulation of autophagy. Human gingiva represents an abundant and easily accessible source of MSCs. The possibility of inducing the differentiation of HGMSCs in an osteogenic sense in vitro can be translated into the regenerative cell therapy of maxillary / mandibular bone defects [5, 6, 48]. Inductive factors such as RV and autophagy modulators can be incorporated into scaffold nanostructures containing HGMSCs and be implanted in situ for repairing and reconstructive purposes) [49].

## Data Availability

All data generated or analysed during this study are included in this research article.

## Abbreviations

COL1A1: Collagen 1α1
HGMSC: Human gingival mesenchymal stem cell
OCN: Osteocalcin
OPN: Osteopontin
RV: Resveratrol
Sp1: Spautin-1
